# Heterologous vaccine interventions: boosting immunity against future pandemics

**DOI:** 10.1186/s10020-021-00317-z

**Published:** 2021-05-31

**Authors:** Daniela Marín-Hernández, Douglas F. Nixon, Nathaniel Hupert

**Affiliations:** 1 Division of Infectious Diseases, Department of Medicine, Weill Cornell Medicine, Belfer Research Building, Room 530, 413 E. 69th street, New York, NY 10065 USA; 2 Department of Population Health Sciences, Weill Cornell Medicine, 402 E. 67th Street, New York, NY 10065 USA; 3 Cornell Institute for Disease and Disaster Preparedness, Weill Cornell Medicine, 402 E. 67th Street, New York, NY 10065 USA

**Keywords:** Vaccine, Non-specific immunity, Innate immunity, Trained immunity, BCG, Influenza vaccine, Vaccination, Heterologous

## Abstract

While vaccines traditionally have been designed and used for protection against infection or disease caused by one specific pathogen, there are known off-target effects from vaccines that can impact infection from unrelated pathogens. The best-known non-specific effects from an unrelated or heterologous vaccine are from the use of the Bacillus Calmette-Guérin (BCG) vaccine, mediated partly through trained immunity. Other vaccines have similar heterologous effects. This review covers molecular mechanisms behind the heterologous effects, and the potential use of heterologous vaccination in the current COVID-19 pandemic. We then discuss novel pandemic response strategies based on rapidly deployed, widespread heterologous vaccination to boost population-level immunity for initial, partial protection against infection and/or clinical disease, while specific vaccines are developed.

## Background

Since the introduction of the smallpox vaccine in 1796, and the Bacillus Calmette-Guérin (BCG) vaccine in 1921, vaccination-related reductions in non-specific morbidity and mortality have been described. Carl Näslund was the first to establish the concept of non-specific immunological vaccine effects, also known as heterologous effects of vaccination (HEV), when BCG vaccination started in Sweden (Aaby and Benn 2019). Subsequent observational studies in areas with high childhood mortality suggested an important reduction in all-cause neonatal mortality and a better survival in early childhood in vaccinated individuals (Garly et al. 2003; Roth et al. 2005). BCG vaccine administered during childhood has been associated with lower mortality from natural causes during almost 40 years of follow-up (Rieckmann et al. 2017a, b). In Japan, people older than 65 with a positive tuberculin test result (from previous BCG vaccination) had a decreased risk of pneumonia versus those not vaccinated (Ohrui et al. 2005). These observations were later corroborated in randomized controlled trials and suggested that the mortality reduction after BCG vaccination was due to the induction of protection against unrelated infectious agents (Aaby et al. 2011).

Measles vaccination (MV) can also have non-specific effects. After the introduction of MV in Guinea-Bissau, there was a significant decrease in childhood mortality (Aaby et al. 1984). Systematic reviews have found evidence suggesting that receiving MV reduced overall mortality by more than would be expected from specific effects alone (Higgins et al. 2016). In high-income countries, the first and second dose of the measles-mumps-rubella (MMR) vaccine was associated with reduction in the risk of hospital admissions, mainly for respiratory infections and long-lasting admission (Sørup et al. 2014, 2019). In the United States, children who had received a live vaccine (mostly MMR) as their most recent vaccination had a lower risk of nontargeted infectious disease hospitalizations (Bardenheier et al. 2017).

Later, attention shifted to another live-attenuated vaccine, the oral polio vaccine (OPV). Since its introduction in South America, reports revealed an interference with the replication of other enteroviruses, resulting in fewer diarrheal deaths (P. Aaby and Benn 2019). Studies in Guinea-Bissau and Ghana associated OPV with up to a 30% reduction in overall mortality rate (Aaby et al. 2004; Welaga et al. 2018). Boosting enhances this beneficial heterologous effects, with each subsequent dose of OPV reducing mortality by an additional 13% (Andersen et al. 2018). While the majority of evidence comes from observational studies, a WHO commissioned review on this topic to evaluate potential bias concluded that BCG and measles-containing vaccines reduced overall mortality by more than would be expected through their effects on the diseases they prevent (Higgins et al. 2016) (Table 1).

**Table 1:** Heterologous effects of vaccination

Vaccine	Heterologous or non-specific effects	References
Vaccinia	Positive effects in papillomas, chronic skin disorders, measles, scarlet fever, whooping cough and syphilis. Reduced risk of infectious disease hospitalization. Protection against HIV-1.	(Mayr 2004) (Sørup et al. 2011) (Weinstein et al. 2010)
BCG	Decreased risk of sexually transmitted HIV-1. Reduced childhood mortality due to respiratory infections and sepsis Reduction in pneumonia and in acute upper respiratory tract infections Protection against influenza virus, yellow fever virus, herpes simplex viruses, respiratory syncytial virus, human papilloma virus, Staphylococcus aureus, Salmonella enteritidis, Yersinia pestis, Klebsiella pneumoniae and Schistosoma mansoni.	(Rieckmann et al. 2017) (Higgins et al. 2016) (De Castro et al. 2015) (Ohrui et al. 2005) (Wardhana et al. 2011) (Moorlag et al. 2019) (Blok et al. 2015)
Measles	Reduced childhood mortality from infectious causes. Reduced nasopharyngeal carriage of Streptococcus pneumoniae and H. influenzae	(Aaby et al. 1995) (Bottomley et al. 2015)
Oral polio	Reduced childhood mortality from infectious causes.	(Andersen et al. 2018)
Inactivated Influenza	Protection against parainfluenza, respiratory syncytial virus and non-influenza virus coinfections.	(Wolff 2020)
Yellow fever	Reduced nasopharyngeal carriage of Streptococcus pneumoniae and H. influenzae.	(Bottomley et al. 2015)

One widely cited “exception” to this pattern of protection is the isolated finding by Benn and Aaby of increased overall mortality in girls in Guinea Bissau who missed one or more of the three-shot diphteria-tetanus-pertussis vaccination series before the age of 18 months; since catch-up on these shots sometimes occurs later after measles vaccination, this report has been taken as cautionary regarding heterologous interaction (i.e., postulating that DPT given after measles vaccination may be deleterious for girls), but the primary data in fact only supports the conclusion of increasing risk with decreasing number of DPT innoculations.

Until recently, the lack of studies evaluating the underlying mechanisms of HEV has been a major obstacle in recognizing and taking advantage of these effects during a pandemic. In this mini-review, we review the immunological mechanisms and the development of the idea of Heterologous Vaccine Intervention (HVI), referring to the exploitation of heterologous or non-specific effects of current approved vaccines against one or more unrelated pathogens. This novel pandemic “early response” intervention, that could be rolled out in parallel with non-pharmaceutical interventions (NPIs) could be used for the current COVID-19 pandemic and for future pandemics to come.

### Innate immune related mechanisms

The long-term functional reprogramming of myeloid cells (monocytes, macrophages, dendritic cells and granulocytes) after a primary exposure, such as an infection or vaccination, that leads to an altered response towards a second exposure after the return to a non-activated immune state, is termed ‘trained immunity’(Netea et al. 2020). This leads to a more effective heterologous (non-specific) response to a subsequent trigger, independent of the initial antigen (Netea et al. 2011). Mechanistically, trained immunity possesses three hallmarks: metabolic, immunological, and epigenetic. The metabolic shift from oxidative phosphorylation towards aerobic glycolysis influences the epigenetic rewiring of innate immune cells, stimulating then the production and release of proinflammatory cytokines, such as TNF-α, IL-6 and IL-1β, and reactive oxygen species upon a second stimulus (Sánchez-Ramón et al. 2018). These features challenge the classical dogma that immunological memory exists only in adaptive immunity, since trained immunity allows the innate immune cells to be ready to react. Animal studies showing protective effects from BCG vaccination against a range of pathogens (Freyne et al. 2015) appear to implicate the induction of myeloid cell differentiation at the level of the hematopoietic stem cell within the bone marrow (Mitroulis et al. 2018)). This has provided justification for studies in humans. For example, using a human in vivo vaccination model, Cirovic et al. recently concluded that BCG vaccination of healthy individuals induces persistent innate immune training and epigenetic changes in peripheral CD14^+^ monocytes and imprints a persistent transcriptomic myeloid bias on human hematopoietic stem and progenitor cell compartment in the bone marrow (Cirovic et al. 2020).

Other innate immune cells that can contribute to protective HEVs include NK cells by increased cytokine production (Rozot et al. 2020; Prabowo et al. 2019) and neutrophils, either due to sufficient numbers of cells secondary to emergency-induced granulopoiesis (Brook et al. 2021) or due to increased antimicrobial activity (Simone et al. 2020). Another cell type which could also be involved are innate-like T cells, specifically MAIT cells that can produce IFN-γ upon BCG induction (Suliman et al. 2019).

Less well studied mechanisms in BCG and other live vaccines are from viral interference (Seppälä et al. 2011), long lasting type I interferon response and antiviral state (Chumakov et al. 2020). Even inactivated vaccines, such as the inactivated influenza vaccine (IIV) has been found to have heterologous effects: the quadrivalent inactivated influenza vaccine induced a trained immunity response after stimulation with a different agent in an in vitro model (Debisarun et al. 2020).

### Adaptive immune related mechanisms

In addition to the effect of a heterologous vaccine on innate immune mechanisms, vaccines can also alter adaptive immune responses to unrelated pathogens and antigens. Two different mechanisms have been described: cross-reactivity and bystander activation. The classical adaptive immune response corresponds to T cells that respond to antigen presentation, but these T cells may cross-react with a different antigen with amino acid similarity (Frankild et al. 2008). In this case of cross-reaction, the T cell would be the same as the involved in the classical response. This is different during bystander activation, in which the response is performed by a neighboring, non-relevant T cell with a specificity that is different from the one involved in the classical response. Such T cells are not activated by T cell receptor ligation but rather via cytokines, such as IL-2, derived from the ongoing response directed against the vaccine-antigen or adjuvant (van Aalst et al. 2017), which increases response of unrelated plasma cells (Bernasconi et al. 2002).

An illustrative example of cross-reactivity resulting from molecular mimicry is the existence of memory T cells specific to viral antigens in unexposed adults, such as herpes simplex virus, cytomegalovirus and HIV-1, possibly as a consequence of cross-reactivity with antigens in the environment. Furthermore, the seasonal influenza vaccine can stimulate T cells specific for a cross-reactive bacterial homologue (Su et al. 2013). In contrast, increased levels of antibodies against measles and *Toxoplasma gondii* in adults are stimulated through bystander activation, upon immunization with tetanus toxoid (Goodridge et al. 2016).

The durability of the heterologous effects after vaccination is unknown, although vaccinia and BCG vaccinations are associated with better long-term survival during a 40-year follow up (Rieckmannet al. 2017a, b). A randomized controlled trial of BCG given at birth led to a reduction in all-cause mortality of 58%, 48% and 21% over the first few days, first month and first year of life, respectively (Biering-Sørensen et al. 2012). In the case of MV the heterologous protection was also higher during the first weeks after vaccination (Aaby et al. 2010). An epidemiological study performed over a 15 year time period reported 70% fewer hospitalizations due to respiratory infections in children 10–14 years old who had received BCG vaccine at birth (De Castro et al. 2015). Kleinnijenhuis et al. found that the long-term heterologous effects, including innate and adaptive immune responses of BCG vaccination in healthy volunteers against non-mycobacterial stimulation, can extend up until 1 year after vaccination (Kleinnijenhuis et al. 2014). Since the long-term effects of vaccines have not been tested in randomized trials, the beneficial effect could be due to unmeasured confounders.

### Heterologous vaccination and the COVID-19 pandemic

Several ecological studies have suggested a negative association between different vaccines and the prevalence or mortality of COVID-19. Escobar et al. attempted to mitigate potential confounding factors and reported a strong correlation between their BCG index and COVID-19 mortality in different socially similar European countries, specifying that every 10% increase in the BCG index was associated with a 10.4% reduction in COVID-19 mortality (Escobar et al. 2020). A exploratory analysis of immunization records found that polio, Haemophilus influenza type-B, measeles-mumps-rubella, Varicella, pneumococcal conjugate, Influenza and hepatitis A/B vaccines administered in the past 1,2 and 5 years were associated with decreased SARS-CoV-2 infection rates (Pawlowski et al. 2021).

An inverse association between influenza vaccination coverage rate in the elderly, at a county or region level in the US (Zanettini et al. 2020) and Italy (Marín-Hernández et al. 2020), and deaths attributable to COVID-19 has been reported. Amato et al. extended these observations by adjusting for potential confounders, concluding that influenza vaccination coverage rate is also independently associated with the SARS-CoV-2 seroprevalence and COVID-19 severity (rates of hospital and intensive care unit admissions) (Amato et al. 2020).

Using the quadrivalent inactivated influenza vaccine applied in the Netherlands in the 2019–2020 influenza season, Debisarun et al. demonstrated the induction of trained immunity response in an in vitro model, including improvement of cytokine responses, after stimulation of human immune cells with SARS-CoV-2 (Debisarun et al. 2020). Additionally, pre-existing SARS-CoV-2 T cells (Grifoni et al. 2020) and SARS-CoV-2 specific antibodies (Ng et al. 2020) have been observed in healthy unexposed donors, which suggests immune cross-reactivity probably among seasonally spreading human coronaviruses, that could provide transient cross-protection.

The concept of Heterologous Vaccine Intervention is being developed with BCG vaccination, and several randomized trials are underway to study if BCG vaccination reduces the incidence or severity of COVID-19 in different countries (NIH U.S. National Library of Medicine 2021). An interim analysis from the randomized clinical trial for enhanced trained immune responses through BCG vaccination to prevent infections of the elderly (ACTIVATE) was published, showing that BCG vaccination in the elderly is safe, significantly increased the time to first infection, and decreased the incidence of new infections, particularly respiratory tract infections of probable viral origin (Giamarellos-Bourboulis et al. 2020). Other explanations should be considered for this negative correlation: the uptake of vaccines might occur in socioeconomic groups with better overall health; alternatively, people who decide to receive vaccines may be more prone to adhere to more stringent physical and social distancing practices.

## Conclusions

Although specific COVID-19 vaccines have been released and given emergency use approval, they are still in limited supply and the slow pace of COVID-19 vaccination is a global challenge. Public health policies against pandemics may derive benefit from the concept of HVI, authorizing the early use of non-specific vaccines either generally as a boost, second dose after the COVID-19 vaccine prime (Hupert et al. 2021) or even using them as vectors for recombinant vaccines (Gupta 2020), achieving a potent protection against COVID-19 with a known safety profile. HVI could be an advantageous option for populations in which effective antibody response to vaccination is known to be suboptimal (e.g., patients with altered immunocompetence) (CDC 2021). The results of a randomized, placebo-controlled pilot study support this idea, showing that BCG vaccination prior to influenza vaccination resulted in a more pronounced and accelerated induction of functional antibody responses and regulates heterologous effects of influenza vaccination with modulation of cytokine responses against unrelated pathogens (Leentjens et al. 2015). Additionally, in an in vitro trained immunity model, the combination of BCG with influenza vaccine induced higher immunological responses upon restimulation with SARS-CoV-2 (Debisarun et al. 2020). The concept of HVI should be considered for future pandemics to bridge the period until a specific vaccine for the disease is developed and globally administered.

## Data Availability

Not applicable.
